# Development of the Digital Health Literacy Instrument: Measuring a Broad Spectrum of Health 1.0 and Health 2.0 Skills

**DOI:** 10.2196/jmir.6709

**Published:** 2017-01-24

**Authors:** Rosalie van der Vaart, Constance Drossaert

**Affiliations:** ^1^ Unit of Health, Medical and Neuropsychology Faculty of Social and Behavioural Sciences Leiden University Leiden Netherlands; ^2^ Department of Psychology, Health and Technology Faculty of Behavioural, Management and Social Sciences University of Twente Enschede Netherlands

**Keywords:** digital health literacy skills, eHealth literacy, measurement, validity, performance-based instrument

## Abstract

**Background:**

With the digitization of health care and the wide availability of Web-based applications, a broad set of skills is essential to properly use such facilities; these skills are called digital health literacy or eHealth literacy. Current instruments to measure digital health literacy focus only on information gathering (Health 1.0 skills) and do not pay attention to interactivity on the Web (Health 2.0). To measure the complete spectrum of Health 1.0 and Health 2.0 skills, including actual competencies, we developed a new instrument. The Digital Health Literacy Instrument (DHLI) measures operational skills, navigation skills, information searching, evaluating reliability, determining relevance, adding self-generated content, and protecting privacy.

**Objective:**

Our objective was to study the distributional properties, reliability, content validity, and construct validity of the DHLI’s self-report scale (21 items) and to explore the feasibility of an additional set of performance-based items (7 items).

**Methods:**

We used a paper-and-pencil survey among a sample of the general Dutch population, stratified by age, sex, and educational level (T1; N=200). The survey consisted of the DHLI, sociodemographics, Internet use, health status, health literacy and the eHealth Literacy Scale (eHEALS). After 2 weeks, we asked participants to complete the DHLI again (T2; n=67). Cronbach alpha and intraclass correlation analysis between T1 and T2 were used to investigate reliability. Principal component analysis was performed to determine content validity. Correlation analyses were used to determine the construct validity.

**Results:**

Respondents (107 female and 93 male) ranged in age from 18 to 84 years (mean 46.4, SD 19.0); 23.0% (46/200) had a lower educational level. Internal consistencies of the total scale (alpha=.87) and the subscales (alpha range .70-.89) were satisfactory, except for protecting privacy (alpha=.57). Distributional properties showed an approximately normal distribution. Test-retest analysis was satisfactory overall (total scale intraclass correlation coefficient=.77; subscale intraclass correlation coefficient range .49-.81). The performance-based items did not together form a single construct (alpha=.47) and should be interpreted individually. Results showed that more complex skills were reflected in a lower number of correct responses. Principal component analysis confirmed the theoretical structure of the self-report scale (76% explained variance). Correlations were as expected, showing significant relations with age (ρ=–.41, *P*<.001), education (ρ=.14, *P*=.047), Internet use (ρ=.39, *P*<.001), health-related Internet use (ρ=.27, *P*<.001), health status (ρ range .17-.27, *P*<.001), health literacy (ρ=.31, *P*<.001), and the eHEALS (ρ=.51, *P*<.001).

**Conclusions:**

This instrument can be accepted as a new self-report measure to assess digital health literacy, using multiple subscales. Its performance-based items provide an indication of actual skills but should be studied and adapted further. Future research should examine the acceptability of this instrument in other languages and among different populations.

## Introduction

Digitization in health care has changed rapidly over the last decades, and online information and (mobile) applications are playing a growing role in health care. Along with these changes, skills to search, select, appraise, and apply online health information and health care-related digital applications are becoming increasingly important for health care consumers. These skills are called digital health literacy [1], or eHealth literacy [2]. The relevance of this form of literacy is demonstrated in recent studies, showing that people’s self-perceived skills to use online information actually affect their health and the quality of their health care, and that a lack of such skills may lead to adverse outcomes [3,4]. Hsu et al. [3] found that digital health literacy skills are associated with various types of health behavior, including healthy eating, exercise, and sleep behavior. Neter and Brainin [4] found relationships between digital health literacy and the presence of chronic illness, perceived self-management skills, and better self-perceived understanding of health status, symptoms, and optional treatments.

A valid measurement instrument on digital health literacy is essential to examine the effects of these skills, both on an individual level and on a population level. On an individual level—for example, in daily clinical practice—a measurement tool could support decisions about the extent to which a patient is able to benefit from particular eHealth tools and interventions [5,6]. Also, it could provide input to coach and train patients who need support in using Web-based health tools [7,8]. On a population level, a proper measurement instrument could provide insight into vulnerable subgroups that face additional challenges in using health care, due to its digitization. For example, previous studies have shown that digital health literacy is related to sociodemographics such as age, education, and income [4,9,10], and studies have shown that certain populations do not have the skills and knowledge to use Web-based health tools for their own benefit and might thereby even become underserved [5,10]. Better insight into populations at risk of low digital health literacy can lead to development and tailoring of health technologies for these specific groups [5,11].

In research, the focus regarding digital health literacy has mainly been on the use of health *information* that is available on the Internet (Health 1.0). Yet eHealth is a broad concept that extends beyond the use of information alone. More recent applications (so called Health 2.0 applications) offer all sorts of interactive technologies, which support people to communicate about their health (with peers and with health care professionals; eg, via forums or e-consults), to self-monitor their health (eg, via patient portals), and even to receive treatment via the Internet (eg, via Web-based cognitive behavioral therapy) [12]. To measure peoples’ ability to use this broad spectrum of applications, an assessment of very diverging skills is essential, since using interactive Health 2.0 applications asks for a more diverse range of skills than retrieving health information alone does [6,13-15]. A study on the digital health literacy skills of patients with rheumatic diseases found that 6 types of competences are essential to properly use both Health 1.0 and Health 2.0 applications [16]. First, people need operational and navigation skills to use a computer and Internet browser; this involves, for example, using a keyboard, touch screen, and search engine and being able to find one’s way around on the Internet. Second, they need information and evaluation skills to search, appraise, and apply online information; this involves, for example, formulating a correct search query, choosing a reliable search result, understanding the obtained search results, and being able to select the results that are reliable and applicable. To use Health 2.0 applications, people need additional skills related to interactivity on the Web. This encompasses adding self-generated content to the Internet (eg, being able to express oneself in written language) and considering both their own and others’ privacy (eg, knowing who is able to read what one has posted on the Internet) [15,16]. Therefore, when measuring a person’s digital health literacy skills, the ability to interact on the Internet should be taken into account as well.

Studies on digital health literacy up until now have used the 8-item eHealth Literacy Scale (eHEALS) [17], which has been the only validated instrument on these skills for a long time. It provides a reliable insight into the self-reported skills of health care consumers when searching and using online health information. Studies on its validation have shown that it measures 1 overall concept [17,18], or 2 separate concepts: seeking and appraising online information [19,20]. In order to extent the measurement of digital health literacy and to assess the broad spectrum of skills that are involved, we developed a new instrument. The Digital Health Literacy Instrument (DHLI) aims to incorporate the diversity of skills to use both Health 1.0 and Health 2.0 tools [14,16]. To promote the feasibility of assessment, this is done with self-reportage of health care consumers’ perceived skills. Nevertheless, it its known that self-reportage can cause a bias, since people tend to over- or underestimate their own Internet skills [18,21,22]. A study on the predictive validity of the eHEALS has shown that the relationship between people’s own perceived skills and their actual performance on Web-based health-related assignments is only small [18]. To overcome this bias in the DHLI instrument, we strive to measure digital literacy skills more objectively as well.

This study’s objective was to determine the instrument’s reliability and validity, and to explore the value of both the self-report items and the performance-based items. To this extent, we determined distributional properties, internal consistency, test-retest reliability, content validity, and construct validity. The construct validity was assessed by studying the correlation with several concepts that can be assumed to be related. First, we investigated the relation with traditional “digital divide” variables (sociodemographics, Internet use, and use of Web-based health apps). Based on previous studies on health literacy and eHealth literacy, we hypothesized small to moderate (.10-.30) negative correlations with age and positive correlations with education and (health-related) Internet use [23-27]. Second, we studied the relation with health status, as digital health literacy can be assumed to have an important influence on health behavior and health-related choices that people make [3,4]. Due to the low number of studies on this subject, and heterogeneity in how health is measured, the expected correlation needs to be estimated. Taking the broadness of this concept into account and all the other variables that influence it, we expected a small correlation of .20. Third, we measured the relation with existing instruments that measure strongly related concepts, namely the Newest Vital Sign (NVS) [28] and the eHEALS [17]. The NVS aims to measure skills related to health literacy (reading ability, numeracy, and applying information). Since this only implies regular health information and does not include digital skills, we expected a moderate correlation (±.30). The eHEALS measures digital health literacy skills, but only on a Health 1.0 level. It does not assess interactive skills on the Internet; therefore, we expected a moderate to large correlation (±.50).

## Methods

### Development of the Digital Health Literacy Instrument

The DHLI operationalizes 7 separate skills. The types of skills are based on a study in which patients with rheumatic diseases were asked to perform a wide range of Health 1.0 and 2.0 eHealth assignments (to find and appraise online health information, to use interactive apps to communicate with peer patients, and to use a personal electronic medical record to retrieve disease-related information and monitor their health status). Since that study used a bottom-up method to determine all relevant skills in health-related use of the Internet, this provided a valid starting point for the instrument [16]. While participants were performing these assignments, we recorded a diverse range of problems, which we divided into 6 categories: (1) operational skills, to use the computer and Internet browser, (2) navigation skills, to navigate and orientate on the Web, (3) information searching skills, to use correct search strategies, (4) evaluating reliability and relevance of online information, (5) adding self-generated content to Web-based apps, and (6) protecting and respecting privacy while using the Internet. In designing the instrument, for each skill we formulated 3 items (in Dutch) to measure people’s self-perceived abilities. In the operationalization process, we divided category 4 into 2 separate concepts—evaluating reliability of the information in general, and determining relevance of the information to oneself in a particular situation—resulting in a total of 7 skill categories measured by 21 self-report items. With these self-report items, people score how difficult they perceive certain tasks to be and how often they experience certain problems on the Internet. Each item was scored on a 4-point scale, with response options ranging from “very easy” to “very difficult” and from “never” to “often.” Scores were reversed, so that a higher score represented a higher level of digital health literacy. The 3 items on the skill of protecting privacy were not obligatory to fill in: when respondents did not have any experience with posting messages on social media or other communication portals, they could leave the items blank.

The DHLI was translated into English, using forward and backward translation, according to World Health Organization guidelines [29]. The exact wording of the items can be found below. We calculated subscores for each skill by using the mean of the 3 items on every skill. We calculated a total score by using the total mean, for which answers on at least 18 items were necessary. Additionally, for each skill, we added a performance-based item, using questions that asked the participant to apply the particular skill in a fictional situation (see Multimedia Appendix 1). Typically, the skill items display a “print screen” of a search engine or website and ask the participant a skill-related question that can be answered that can be scored as correct (score=1) or false (score=0). Examples of performance-based questions are what button to press for a certain action, or what piece of information would be most valuable in a certain situation. Each item has 5 answer options: 4 different answers (of which 1 is correct) and an “I don’t know” option (score=0). Each correct answer receives 1 point, adding up to a maximum total score of 7 points. To calculate a total score, at least 6 out of 7 items should be answered.

We tested face validity of this initial instrument among 11 people, using a 3-step test cognitive interview [30]. Participants were asked to think aloud while completing the items, in order to gain insight into their reasoning and decision-making process when answering the questions [31]. After completion, the research leader asked several follow-up probing questions related to the items that had seemed to cause problems in understanding or answering. In this way, we gained insight into the readability and clarity of the items and altered them accordingly. After these initial alterations, we conducted a second pilot test among 8 people. We made only a few minor alterations in wording in this last pilot round.

### Design of the Survey Study

We studied the reliability and validity of the instrument in a paper-and-pencil survey study among the general Dutch population. We did not use a Web-based survey, in order not to exclude people with low digital health literacy skills beforehand.

### Participants and Procedure

A total of 200 people participated in the study. Inclusion criteria were having Internet access, being fluent in Dutch, and being 18 years of age or older. We recruited participants through convenience sampling using stratification based on age, sex, and educational level to reach an equal distribution on these sociodemographics. Regarding age, the categories were (1) 18-34 years, (2) 35-49 years, (3) 50-64 years, and (4) 65 years or older. Regarding education, the categories were (1) low: no education, primary school only, or lowest level of high school, (2) middle: higher levels of high school or secondary vocational education, and (3) high: bachelor’s degree or higher. On this variable, complete stratification was not feasible, resulting in an overrepresentation of more highly educated respondents.

People who were invited to participate received an invitation letter explaining the inclusion criteria, purpose of the study, its duration (30 minutes), and its voluntary nature. People who consented to take part in the study were contacted in person, by telephone or email, to confirm their interest in the study and to schedule an appointment. The assessment was done at a quiet location (mostly the participant’s home). At the start of the survey (T1) an informed consent form was signed. Participants were asked to fill out the questionnaire and, after that, the research leader assessed the NVS (see Measures section) in a face-to-face setting, which took approximately 4 minutes. We asked all participants 2 weeks later to fill out the DHLI again (T2). After completion of data collection, we raffled off 10 gift certificates of €25 each among the participants at T1.

The study was approved by the Psychology Ethics Committee of Leiden University, Leiden, the Netherlands.

### Measures

Besides the DHLI, the survey assessed the participants’ (1) sociodemographics: sex, age, and educational level; (2) Internet use: means of Internet access, frequency of Internet use, and self-rated Internet skills; (3) health-related Internet use; (4) health status; (5) health literacy; and (6) eHealth literacy.

We measured health-related Internet use by asking participants the number of occasions on which they had used several eHealth applications, divided into online information, health-related communication tools (such as a patient forum and e-consult), and treatment-related applications (monitoring, Web-based self-help, mobile phone app), with a total of 12 items. Answer options were “never” (score=0), “once” (score=1), “several times” (score=2), and “often” (score=3). We calculated the sum score by adding up the scores on each item.

We measured health status with 3 subscales of the Dutch version of the RAND 36-Item Health Survey (RAND-36), namely General Health Perceptions, Physical Functioning, and Emotional Well-being [32-34]. These scales contain, respectively, 5, 10, and 5 items on perceived general health and perceived health in relation to others (alpha=.81), experienced limitations due to physical health (alpha=.92), and states of emotional well-being (alpha=.85) [34].

We measured health literacy with the Dutch version of the NVS [28,35]. The instrument consists of 6 items based on a nutrition label from an ice cream container. The NVS measures reading skills, numeracy skills, and the ability to apply information. Each correctly answered item receives 1 point, which can be summed up as a total sum score (alpha=.78).

We measured eHealth literacy with the Dutch version of the eHEALS [17,18]. The eHEALS contains 8 items on self-perceived skills to use online health information, measured by a 5-point Likert scale with response options ranging from “strongly disagree” to “strongly agree.” Total scores of the eHEALS are summed to range from 8-40, with higher scores representing higher self-perceived eHealth literacy (alpha=.93).

### Data Analyses

Data were analyzed using IBM SPSS version 23.0 for Windows (IBM Corporation). Cronbach alpha served as a measure of internal consistency, reflecting the (weighted) average correlation of items within the scale [36]. In general, a Cronbach alpha of .7-.8 is regarded as satisfactory for scales to be used as research tools [37]. We calculated item-total correlations using Spearman rho correlations. Distributional properties of the DHLI and the possible subscales were inspected to examine their normality and to identify floor and ceiling effects. We used skewness and kurtosis values, as well as a Kolmogorov-Smirnov test, to assess the distribution of the scores at T1 and T2. Skewness and kurtosis scores between ±1 and significance on the Kolmogorov-Smirnov test indicate no or slight nonnormality [38]. We considered floor or ceiling effects to be present if >15% of the participants scored the worst or the best possible score on the subscales [39]. Paired samples *t* tests were performed to check for any differences between T1 and T2. To study the test-retest reliability, we calculated intraclass correlation coefficients (ICCs). We assumed a correlation of ≥.70 to be satisfactory [40]. Content validity was assessed with a principal component analysis and varimax rotation to examine the fit with the theoretical 7-factor structure of the instrument. We used expectation-maximization imputations for the missing data. The suitability of using factor analysis on the dataset was assessed using Bartlett test of sphericity (*P*<.05) and the Kaiser-Meyer-Olkin statistic (recommended value of .6) [38]. We considered factor loadings in excess of .71 to be excellent, .63 to be very good, and .55 to be good [37]. Evidence for construct validity was determined by studying Spearman rho correlations between total scores on the DHLI and sociodemographics, (health-related) Internet use, health status, the NVS, and the eHEALS.

## Results

### Participants

In total, 200 respondents completed the survey at T1. The response rate on the retest survey was 33.5%; 67 respondents completed the DHLI at T2. Table 1 shows the characteristics of the sample populations at T1 and T2. At T1, 53.5% (107/200) were female. Mean age was 46.4 (SD 19.0) years, and the distribution among the 4 age groups was rather equal, with participants between 18 and 34 years old making up 30.0% (60/200); between 35 and 49, 21.0% (42/200); between 50 and 65, 28.5% (57/200); and 65 and older, 20.5% (41/200). More highly educated people were overrepresented, at 41.5% (83/200) of the total sample.

**Table 1:** Sociodemographics of participants completing the survey at baseline (T1; N=200) and at 2 weeks (T2; n=67).

Characteristics		T1	T2
**Sex, n (%)**			
	Male	93 (46.5)	31 (46)
	Female	107 (53.5)	36 (54)
**Age in years**			
	Mean (SD)	46.4 (19.0)	46.2 (16.3)
	Range	18-84	18-78
**Educational level, n (%)**			
	Low	46 (23.0)	13 (19)
	Middle	71 (35.5)	27 (40)
	High	83 (41.5)	27 (40)

The largest proportion of the respondents used the Internet frequently (see Table 2) and rated their Internet skills as excellent (n=59, 29.5%) or good (n=81, 40.5%). Most respondents accessed the Internet via a mobile phone (n=166, 83.0%), laptop (n=161, 80.5%), personal computer at home (n=115, 57.5%), or tablet (n=113, 56.5%). Of all respondents, 89.5% (n=179) had ever searched the Internet for health- or treatment-related information. Around half had ever read posts on a health-related peer support forum or social media website (n=103, 51.5%) or a health care review website (n=92, 46.0%). A third had ever used a health-related mobile phone app (n=65, 32.5%). Posting self-generated content on the Internet and using treatment-related apps was reported by a smaller proportion of the sample (between 5.5% and 18.0%, see Table 2). Respondents who filled out the survey at T2 did not differ from the total sample on any of the demographic variables, but did report using the Internet more often (*t*_163_=1.30, *P*=.02). This suggests that nonresponse bias might have occurred.

**Table 2:** General and health-related Internet use among respondents at baseline (T1; N=200) and at 2 weeks (T2; n=67).

		T1, n (%)	T2, n (%)
**Frequency of Internet use**			
	(Almost) every day	178 (89.0)	63 (94)
	Several days a week	12 (6.0)	2 (3)
	About 1 day a week	5 (2.5)	1 (2)
	(Almost) never	3 (1.5)	1 (2)
**Means of Internet access^a^**			
	Mobile phone	166 (83.0)	61 (91)
	Laptop	161 (80.5)	57 (85)
	Personal computer at home	115 (57.5)	33 (49)
	Tablet	113 (56.5)	36 (54)
	Computer at work	87 (43.5)	33 (49)
	Public computer	26 (13.0)	10 (15)
**Self-rated Internet skills**			
	Excellent	59 (29.5)	18 (27)
	Good	81 (40.5)	30 (45)
	Average	38 (19.0)	15 (22)
	Reasonable	17 (8.5)	3 (5)
	Poor	5 (2.5)	1 (2)
**Number of respondents who have ever used the Internet to…**			
	Search for information on health or illness	179 (89.5)	57 (85)
	Schedule an appointment with their health care provider	103 (51.5)	36 (54)
	Read on a health-related forum or social media website	103 (51.5)	31 (46)
	Read a health care review	92 (46.0)	35 (52)
	Use a health-related mobile phone app	65 (32.5)	26 (39)
	Ask a question of their health care provider	36 (18.0)	13 (20)
	Monitor disease symptoms	34 (17.0)	10 (15)
	Share personal medical information with others	24 (12.0)	13 (19)
	Log on to their own electronic medical record	14 (7.0)	5 (8)
	Post a health care review	11 (5.5)	5 (8)
	Take a Web-based self-management course	10 (5)	5 (8)
	Post a message on a peer support forum or social media website	9 (4.5)	2 (3)

^a^Respondents could mark more than 1 answer on this item.

### Distributional Properties and Reliability of the Digital Health Literacy Instrument

Table 3 shows the scores and internal consistency of the self-report part of the DHLI. The Cronbach alpha is satisfactory, at .87. The Cronbach alpha of the items on each separate skill are satisfactory as well, indicating that these scales can be used as a subscale in the DHLI (alpha range .70-.89). Only the skill protecting privacy had an unsatisfactory Cronbach alpha score (.57). The item-total correlations (not shown in Table 3) were moderate to large for all items (range .51-.73, *P*<.001), except for the items on the skill protecting privacy, which showed no significant item-total correlation. Respondents had a total mean score of 3.11 (SD 0.87). Total scores were slightly skewed (–1.004) and showed kurtosis (2.251) due to frequent scores between 2.75 and 3.5. However, the Kolmogorov-Smirnov test was not significant (*D*_200_=.06, *P*=.06), indicating that the scores are approximately normally distributed. The highest scores on the subscales were reported on operational skills (mean 3.67, SD 0.59), navigation skills (mean 3.30, SD 0.52), and protecting privacy (mean 3.52, 0.52). Operational skills were strongly skewed (–2,388), with a ceiling effect of 60.0% (120/200) scoring the highest possible score, and showed kurtosis (6.220). Privacy protecting skills were slightly skewed (–1.059), with 16.0% (32/200) scoring the highest possible score and no one scoring the lowest possible score. Since the items of the protecting privacy scale were not obligatory to fill in, the response rate on this scale was lower (n=86). The scores of respondents who completed the DHLI at both T1 and T2 did not differ from the total sample at T1 (test statistics not shown in Table 3). Cronbach alphas of the subscales at T2 were satisfactory, ranging from .68 to .88. The test-retest reliability was satisfactory, with ICC=.77 (*P*<.001) between T1 and T2 on the total scores and levels of agreement of .49-.81 on the subscales.

The Cronbach alpha of the performance-based items was .47, which means that these items did not together form a single construct and should be interpreted as separate items that measure individual skills. Table 4 shows the number of respondents who answered each performance-based item correctly. Most respondents answered the items correctly. Among the more complex skills, the number of respondents with an incorrect answer increased. The only exception was evaluation reliability, which was answered correctly by 94.5% of the respondents (n=188).

**Table 3:** Total scores, subscale scores, and internal consistencies on the Digital Health Literacy Instrument at baseline (T1; N=200) and at 2 weeks (T2; n=67).

Digital health literacy skill	T1 (N=200) mean (SD)	Alpha T1	T1 (n=67)^a^	T2 (n=67) mean (SD)	Alpha T2	ICC^b^ between T1 and T2 (n=67)	*P* value
Total digital health literacy	3.11 (0.45)	.87	3.12 (0.39)	3.16 (0.41)	.88	.77	<.001
Operational skills^c^	3.67 (0.59)	.77	3.76 (0.43)	3.68 (0.51)	.86	.81	<.001
Navigation skills^c^	3.30 (0.52)	.70	3.38 (0.42)	3.28 (0.52)	.82	.60	<.001
Information searching^c^	3.04 (0.64)	.89	3.00 (0.62)	3.00 (0.50)	.82	.63	<.001
Evaluating reliability^c^	2.70 (0.63)	.78	2.74 (0.61)	2.84 (0.53)	.79	.67	<.001
Determining relevance^c^	2.81 (0.60)	.81	2.82 (0.56)	2.85 (0.58)	.85	.49	<.001
Adding content^c^	3.00 (0.67)	.89	2.98 (0.72)	3.14 (0.61)	.91	.58	<.001
Protecting privacy (T1 n=86; T2 n=38)	3.52 (0.52)	.57	3.38 (0.46)	3.61 (0.50)	.68	.49	<.02

^a^Scores at T1 of those who also completed the survey at T2.

^b^ICC: intraclass correlation coefficient.

^c^Answer score range 1-4.

^d^Answer score range 2-4.

**Table 4:** Number and percentages of respondents who answered the performance-based items correctly (n=199).

Subscale	Respondents with correct answer, n (%)
Operational skills	191 (96.0)
Navigation skills	167 (83.9)
Information searching	156 (78.4)
Evaluating reliability	188 (94.5)
Determining relevance	139 (69.8)
Adding content	135 (67.8)
Protecting privacy	111 (55.8)

### Content Validity of the Digital Health Literacy Instrument

Since the performance-based items did not form a scale together, we further determined the content validity of only the self-report scale. Principal component analysis showed a Bartlett test of sphericity of χ^2^_210_=2278.360, *P*<.001, indicating that correlations between items were sufficiently large for this analysis. The correlation matrix showed no correlations higher than .9, indicating an absence of multicollinearity. The Kaiser-Meyer-Olkin measure of sampling adequacy was good (.859), which indicates that the sample size was adequate for factor analysis. Two eigenvalues were lower than 1: navigation skills (0.949) and protecting privacy (0.816). The others exceeded 1, ranging from 1.124 to 7.580. In combination, the scales explained 76% of the variance, varying between 8% and 14% among the subscales. Table 5 shows the factor loadings after rotation. The items clustered among the factors as intended, with satisfactory factor loadings. Only item 9 scored below .55.

**Table 5:** Principal component analysis on the Digital Health Literacy Instrument at baseline (T1; N=200).

Item		Component^a^						
		1	2	3	4	5	6	7
*How easy or difficult is it for you to…*								
1.	Use the keyboard of a computer (eg, to type words)?	.838						
2.	Use the mouse (eg, to put the cursor in the right field or to click)?	.879						
3.	Use the buttons or links and hyperlinks on websites?	.817						
*When you search the Internet for information on health, how easy or difficult is it for you to…*								
4.	Make a choice from all the information you find?		.777					
5.	Use the proper words or search query to find the information you are looking for?		.755					
6.	Find the exact information you are looking for?		.818					
7.	Decide whether the information is reliable or not?			.621				
8.	Decide whether the information is written with commercial interests (eg, by people trying to sell a product)?			.848				
9.	Check different websites to see whether they provide the same information?			.547				
10.	Decide if the information you found is applicable to you?				.557			
11.	Apply the information you found in your daily life?				.777			
12.	Use the information you found to make decisions about your health (eg, on nutrition, medication or to decide whether to ask a doctor’s opinion)?				.824			
*When you search the Internet for health information, how often does it happen that…*								
13.	You lose track of where you are on a website or the Internet?					.705		
14.	You do not know how to return to a previous page?					.584		
15.	You click on something and get to see something different than you expected?					.805		
*When typing a message (eg, to your doctor, on a forum, or on social media such as Facebook or Twitter) how easy or difficult is it for you to…*								
16.	Clearly formulate your question or health-related worry?						.825	
17.	Express your opinion, thoughts, or feelings in writing?						.880	
18.	Write your message as such, for people to understand exactly what you mean?						.891	
*When you post a message on a public forum or social media, how often…*								
19.	Do you find it difficult to judge who can read along?							.797
20	Do you (intentionally or unintentionally) share your own private information (eg, name or address)?							.791
21.	Do you (intentionally or unintentionally) share some else’s private information?							.888
Eigenvalue		7.58	2.16	1.59	0.95	1.12	1.91	0.82
% of variance		14.3	12.5	9.9	9.2	9.6	12.3	8.0

^a^The items were as follows: 1: operational skills; 2: information searching; 3: evaluating reliability; 4: determining relevance; 5: navigation skills; 6: adding self-generated content; 7: protecting privacy.

### Construct Validity of the Self-Report Scale of the Digital Health Literacy Instrument

Table 6 shows the Spearman rho correlations between the total score on the DHLI and the other assessed variables. Overall, age showed a moderate negative correlation, indicating that older age is related to lower digital health literacy. The other variables showed low to high positive correlations, indicating that a higher educational level, Internet use, health-related Internet use, better health status (as measured with the RAND-36), health literacy (as measured with the NVS), and eHealth literacy (as measured with the eHEALS) are related to higher digital health literacy skills.

**Table 6:** Spearman rho correlations between the Digital Health Literacy Instrument, sociodemographics, Internet use, health perception, the NVS^a^ and the eHEALS^b^.

Variable assessed	ρ	*P* value
Age	–.41	<.001
Education	.14	.047
Internet use	.39	<.001
Health-related Internet use	.27	<.001
Health perception (RAND-36^c^)	.23	<.001
Physical functioning (RAND-36)	.27	<.001
Mental well-being (RAND-36)	.17	.047
Health literacy (NVS)	.31	<.001
eHealth literacy (eHEALS)	.51	<.001

^a^NVS: Newest Vital Sign.

^b^eHEALS: eHealth Literacy Scale.

^c^RAND-36: RAND 36-Item Health Survey.

## Discussion

Up until now, measurement instruments on digital health literacy skills have measured only competencies related to searching and using online health information (Health 1.0). No instrument has yet been available that also measures the broader range of skills that are essential to using eHealth applications, including more interactive Health 2.0 skills [17,41]. Moreover, the available instruments are self-report assessments, which provide no information on people’s actual competence level [18]. This paper introduces the newly developed DHLI to assess both Health 1.0 and Health 2.0 skills, using self-reportage and performance-based items.

Our results on the nature and scope of our respondents’ health-related Internet use underscore the need for a broad measurement instrument. Whereas searching for health-related information on the Web was still most common (conducted by >90%), more than half of the respondents also reported using health-related social media or consumer review sites. Looking at the measurement properties of the DHLI, it can be concluded that the instrument indeed measures a wide range of digital health literacy skills. The overall reliability of the self-report scale of the instrument can be concluded to be sufficient, with satisfying Cronbach alpha scores and a proper overall test-retest reliability. Only the results on the skill protecting privacy are less convincing, which indicates that this subscale should be further improved. Furthermore, the content validity is good, with the 7 theoretical subscales represented in 7 separate factors, which together explain the largest part of variance. The distribution of the self-report scale can be assumed to be approximately normal, despite some skewness and kurtosis in the total scale and 2 subscales. People in our sample tended to score mostly in the third and fourth quartile of the answer range, meaning that they perceived their skills to be good to very good.

Among the subscales, operational skills showed a high ceiling effect; the largest proportion of our samples (at T1 and T2) scored the highest possible score on this scale. This indicates that the general population does not experience problems in this area, which is not very surprising because this can be seen as the most basic skill in using the Internet. Nevertheless, from previous studies, it is known that a smaller subgroup in the population does struggle with these skills [14,16], which makes it nonetheless relevant to assess these competencies. Further research needs to consider the instrument’s application to other subgroups for which these skills might be less obvious (such as the elderly and less-educated people) due to less computer experience [7,42]. What is remarkable in relation to the operational skills subscale as well is that the majority of our sample accessed the Internet with a mobile phone, and not with a laptop or personal computer. Operational skills require different competencies, since these devices operate in very diverse ways in terms of knowledge of the function of various buttons, using a cursor, and clicking on items. Therefore, a future developmental step should take this into account and add mobile health skills (mHealth) as well.

In order to measure more than people’s perceived digital health literacy skills, we added a performance-based item to each self-report subscale. Together, the performance-based items showed a low internal consistency, which means that the items should be interpreted individually. The low internal consistency could be explained by the diverse nature of the items. As single items they might be usable to detect specific problems in individuals’ competencies. To test this, further research should determine how applicable these items are among subgroups with low digital health literacy skills and what the discriminant value is among these groups. Possibly, the items should be altered to compose more difficult tasks. In our sample most respondents answered the questions correctly, but the more complex the skill, the larger the proportion of the sample with an incorrect answer. The only exception to this trend was the item on evaluating reliability. We measure this skill by asking the respondent where to check the source of the information on a website. Possibly, this question is too easy and does not represent this skill sufficiently. All in all, these items propose a new method to measure actual digital health literacy skills; from here on their applicability should be improved.

Concerning the validity of the DHLI, the correlations between the self-report scale and related variables were as we expected. The relationship between digital skills and both age and education is still present, possibly due to less computer and Internet use [4,43]. This is confirmed by the positive correlations found between digital skills and both Internet and eHealth use. However, the correlation with education is only low, showing a catch-up in skills by the less educated, narrowing this existing gap. This low correlation might be explained by the high availability of the Internet in general in the Netherlands (Internet access is 92% for less-educated people vs 99% for more highly educated people [44]).

The low, but significant, correlations between health status and digital health literacy indicate a relation between people’s skills in using Web-based health care and their actual health. This is interesting, since it indicates the impact that using eHealth can have on people’s lives. However, no conclusions can be drawn on the causality of this relationship from our data, and the associations found with age and education should be taken into account in this context as well, since these variables are also related to health. Previous studies did find a mediating role of digital health literacy on health behavior [3] and a relationship with self-management of health and interaction with physicians [4]. Future research should reveal more on the impact that digital health literacy has on (physical, mental, and social) health and health behavior, and on how these competencies can be influenced or deployed to improve one’s health.

The correlation between the DHLI and health literacy was moderate, which corresponds with a previous study in which a correlation of *r*=.36 was found between health literacy and digital health literacy [20]. Since digital health literacy comprises both general health literacy and digital skills, a moderate correlation seems appropriate. The correlation with the eHEALS was moderate to high, which shows there is overlap between the 2 instruments, as expected. Still, it also shows that this new instrument partly measures different skills. To further explore the construct validity of the DHLI, we aim to perform follow-up research on the relationship between scores on this instrument and other health-related factors, such as knowledge on health and disease, health behavior, and self-efficacy in health care [3,4,20].

A limitation of this study that should be taken into account is the overrepresentation of more highly educated respondents, which hinders the translation of these results to the general population. Moreover, as stated before, it is particularly interesting to determine the applicability of this instrument among groups at risk for low digital health literacy. This is, therefore, a large implication for further research. A second limitation, related to the performance-based items, concerns the use of 1 format in the formulation of the items. We used print screens from the Web browser Google Chrome; however, naturally many people use other Web browsers and other operating systems (eg, OS X instead of Windows), which intervene with the validity of the items. When the instrument is assessed digitally, an adaptive test could overcome this problem, so participants can first supply information on their browser use, to which the items can be adjusted. With a paper-and-pencil assessment this could also be done when the instrument is used individually (then the suitable version would be handed to the person), but in a (anonymous) research setting, this will be a persistent problem.

All in all, it can be concluded that the DHLI is acceptable as a new measurement tool to assess digital health literacy, measuring 6 diverse skills. Its self-report scale shows proper reliability and validity. The included performance-based items should be studied and adapted further, to determine their value and their discriminant validity. Future research should examine the acceptability of this instrument in other languages and among different (risk) populations and should explore ways to measure mobile health literacy skills as well.

The Digital Health Literacy Instrument, in both Dutch and English, is available and may be used on request via the corresponding author.

## Appendix

Multimedia Appendix 1 English version of the Digital Health Literacy Scale's 7 performance-based items.

## Abbreviations

DHLI: Digital Health Literacy Instrument
eHEALS: eHealth Literacy Scale
ICC: intraclass correlation coefficient
NVS: Newest Vital Sign
RAND-36: RAND 36-Item Health Survey
