# Supplementary material for: Development of the Digital Health Literacy Instrument: Measuring a Broad Spectrum of Health 1.0 and Health 2.0 Skills
Source: J Med Internet Res. 2017 Jan 24;19(1):e27. doi: 10.2196/jmir.6709 (PMC5358017; doi:10.2196/jmir.6709)
Supplement: Multimedia Appendix 1 [file jmir_v19i1e27_app1.pdf]

Multimedia Appendix - Digital Health Literacy Scale performance-based items

Below (figure 1) you see part of the home page of the website www.kiesbeter.nl, a national website which provides information on healthcare and care providers in the Netherlands.

If you were to minimalize this page, to open another program on your computer. Which button would you use?

- ☐ Button 1 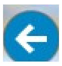
- ☐ Button 2 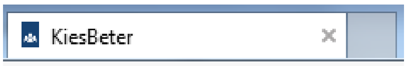

- ☐ Button 3 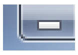
- ☐ Button 4 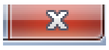
- ☐ I don't know

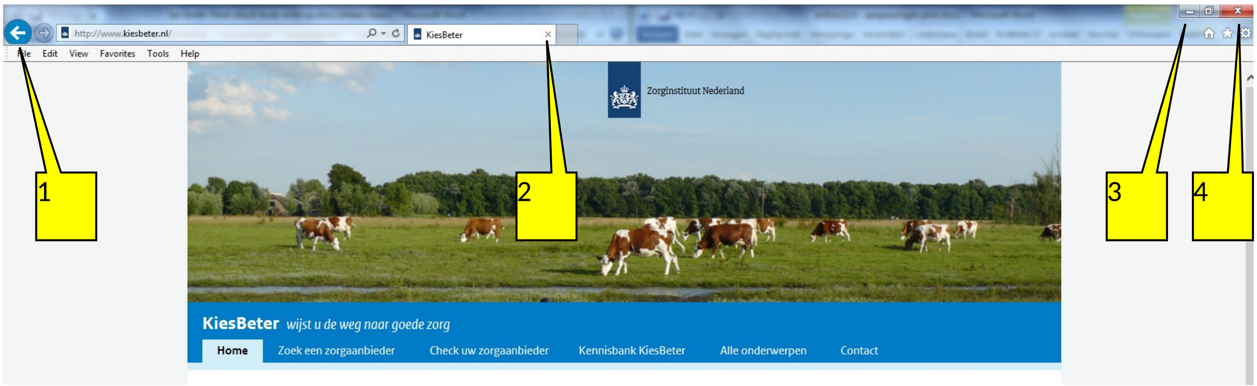

Figure 1

Imagine, you are searching for a general practitioner in your neighborhood. Via Google you found the website www.kiesbeter.nl (see figure 2). Then, you want to leave this website and go back to your search results in Google. Which button would you use?

- ☐ Button 1 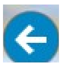
- ☐ Button 2 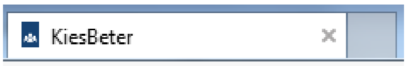

- ☐ Button 3 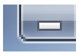
- ☐ Button 4 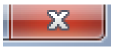
- ☐ I don't know

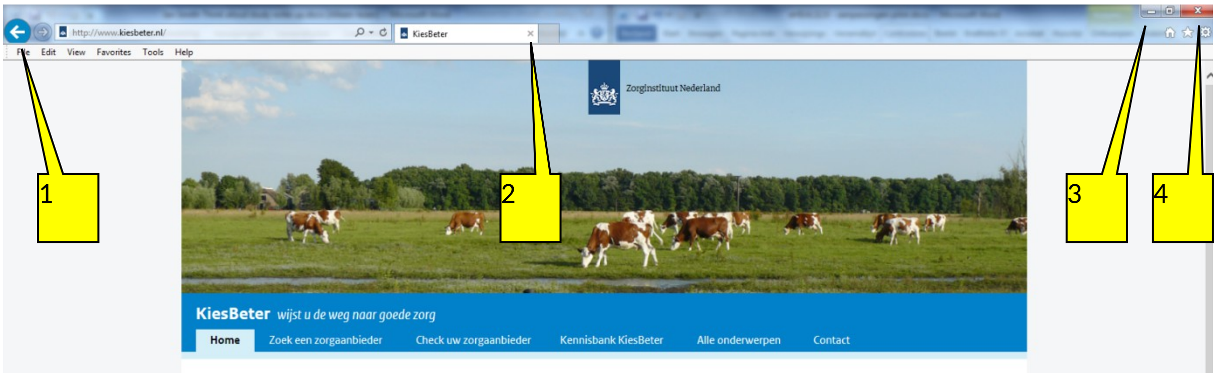

Figure 2

Imagine, you are under treatment for a rheumatic condition at the ReumaCentrum Twente in Enschede, the Netherlands. You visit the department's website (see figure 3). You browse around on the website. What kind of information do you expect to find when you click on button A?

- ☐ Information on rheumatic conditions
- ☐ Information on your personal treatment (such as diagnosis and medication)
- ☐ Information on the department of rheumatology
- ☐ Information on the Dutch Arthritis Association
- ☐ I don't know

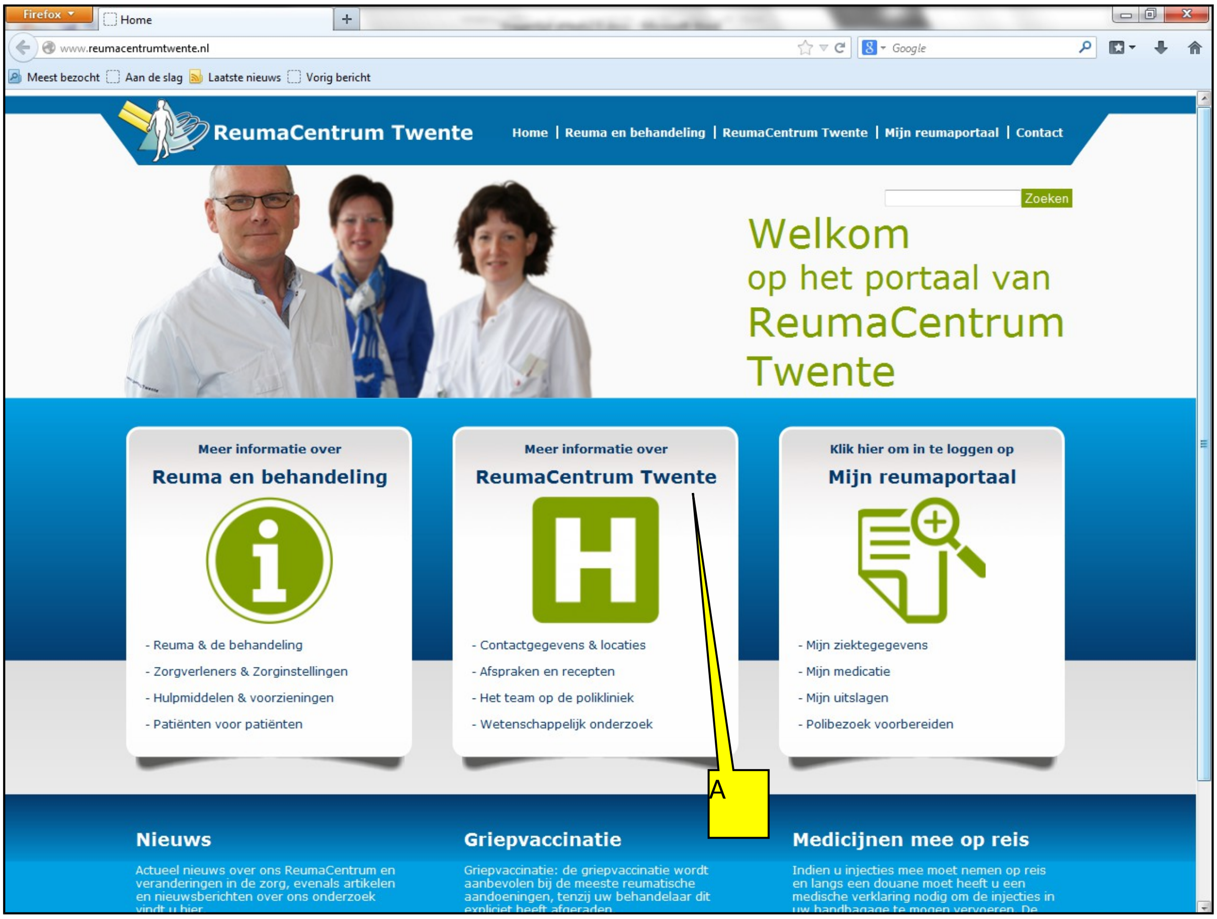

Figure 3

Imagine, you are looking for information on depression. You find the website below, from the Dutch foundation for Psychological Health (see figure 4). You want to know who are behind the foundation. Which button would you click on?

- ☐ Button 1: “Over ons” (“about us”)
- ☐ Button 2: “Contact” (“contact”)
- ☐ Button: “Disclaimer en privacybeleid” (“disclaimer and privacy policy”)
- ☐ Button: “Sitemap”
- ☐ I don’t know

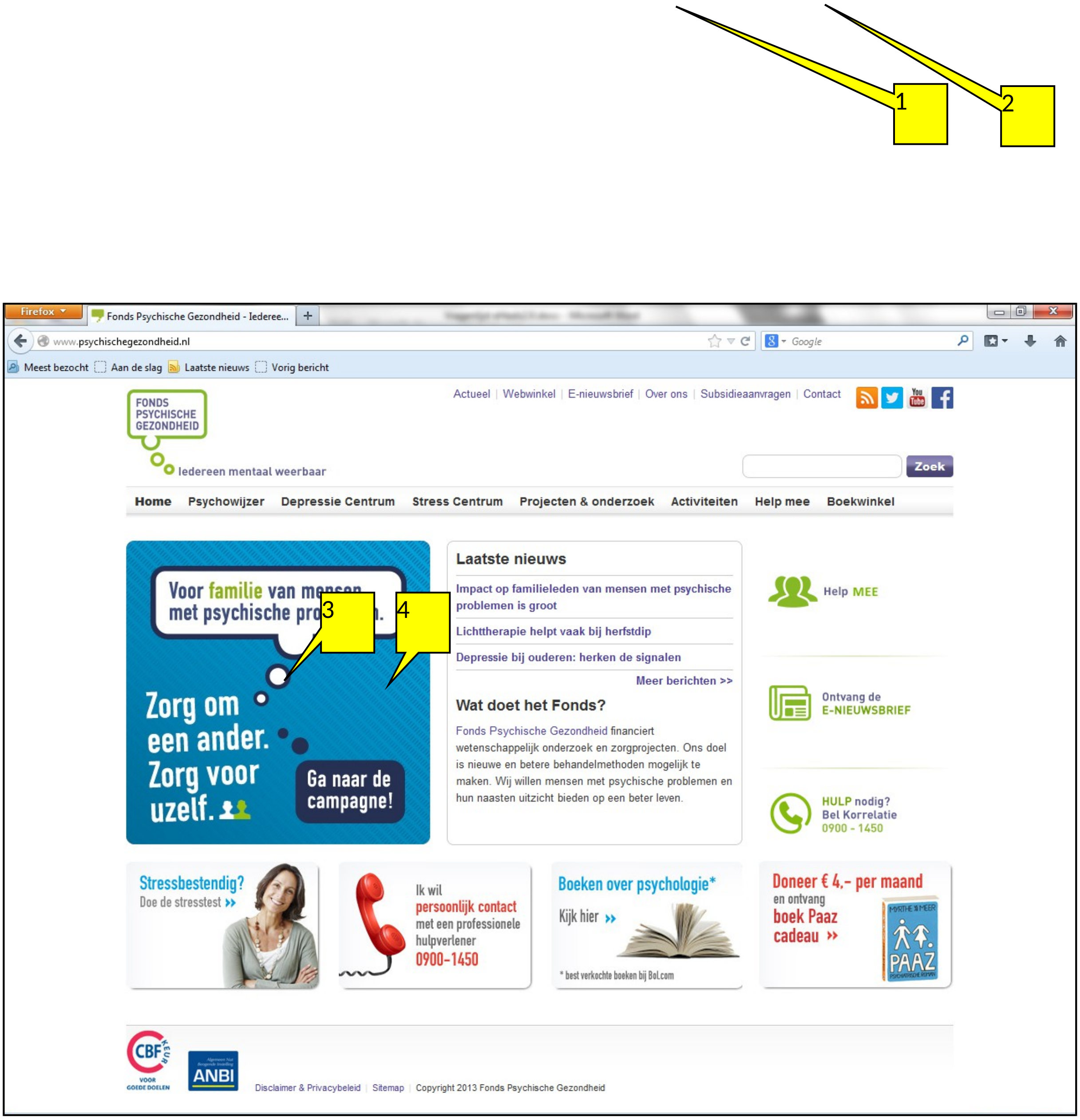

Figure 4

Imagine, your 16 years old child is very fatigued lately. Your neighbor suggested that it might be Pfeiffer's disease ("mono"). You want to find out what the exact symptoms are of Pfeiffer's disease. A search via Google gives you the results below. Which of these results would most likely give you a correct and reliable answer?

☐ Search result 1:

Oorzaak van Ziekte van **Pfeiffer** - Aandoening - Gezondheidsplein.nl
www.gezondheidsplein.nl/aandoeningen/184/ziekte...**pfeiffer/oorzaa**...
Oorzaken van de ziekte van **Pfeiffer**. De **oorzaak** van de ziekte is het Epstein Barr-virus (een herpesvirus). Dit vermenigvuldigt zich in de witte bloedlichaampjes, **...**

[Translation]
Cause of **Pfeiffer's disease** – Disease – Healthsquare.nl
Causes of **Pfeiffer's disease**. The **cause** of the disease is the Epstein Barrvirus (a virus related to herpes). This multiplies in the white blood cells, ...

☐ Search result 2:

Pfeiffer, ziekte van - **Artsennet**
www.**artsennet**.nl/Richtlijnen/Richtlijn/42995/**Pfeiffer**-ziekte-van.htm
Pfeiffer Keelpijn, opgezwollen klieren, koorts, nergens zin in hebben en de hele tijd ontiegelijk moe zijn. De **symptomen** van de **ziekte van pfeiffer**. Een.

[*Translation*]
Pfeiffer's disease – Doctor's net
Pfeiffer's disease, sore throat, swollen glands, fever, not being in the mood for anything, extreme fatigue. The **symptoms** of **Pfeiffer's Disease**.]

☐ Search result 3

Ziekte van **Pfeiffer**: Ik heb de ziekte van **Pfeiffer** | Thuisarts.nl
www.thuisarts.nl/ziekte-van-**pfeiffer**/ik-heb-ziekte-van-**pfeiffer**
1 nov 2011 – Er bestaan geen **medicijnen** tegen de ziekte van **Pfeiffer**. De ziekte gaat vanzelf over, maar hoelang de ziekte duurt, is niet te voorspellen.

[*Translation*]
Pfeiffer's disease: I have **Pfeiffer's disease**. Home doctor.nl
There is no **medicine** available to cure **Pfeiffer's disease**. The illness passes by its self, but how long that takes is unpredictable.

☐ Search result 4:

symptomen van **pfeiffer** - **Forum** - Ziekte van **Pfeiffer**
forum.ziektevanpfeiffer.nl › Ziekte van Pfeiffer › Algemeen
36 berichten - 11 auteurs - 7 feb
Zie hieronder een lijstje van **symptomen** die de meeste leden van dit **forum** hebben of die meerdere leden hebben. Misschien prettig om bij **...**

[*Translation*]
symptoms of **Pfeiffer's disease** – **forum** – **Pfeiffer's disease**
See below a list of **symptoms** that most or many members of this **forum** report. Perhaps it is good to ...

☐ I don't know

Here below you find three messages from patients, or their relatives, on a discussion forum. Which of the messages takes privacy into account properly?

- ☐ Message 1 (from Aline)
- ☐ Message 2 (from Silvia)
- ☐ Message 3 (from Annelies)
- ☐ I don't know

<table>
<tr>
<td>Re: Hoe het gaat........

door Aline » do okt 16, 2014 7:32 pm

hoi

ik had ook mtx spuiten en ben daar mee gestopt ik heb nog wel de enbrel spuiten en daar heb ik geen last van bij mij werkt het prima, hopelijk slaat het bij jou ook aan en voel je , je niet ziek daarvan, wens je een hele fijne vakantie.

groetjes Aline</td>
<td>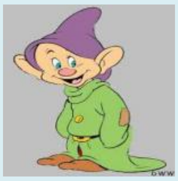
Aline
Lid

Berichten: 452
Woonplaats: Rotterdam
Type Reuma:
Reumatoide Arthitis</td>
</tr>
<tr>
<td>[Translation]

Re: How I am doing…….

by Aline » Thursday Oct 16, 2014 7.32 PM

Hi

I also used MTX injections and I quit with those I do still have Enbrel injections and they don't bother me they work fine for me, I hope it catches on for you as well and it won't make you sick, I wish you a very pleasant vacation.

Regards Aline</td>
<td>Aline
Member

Messages: 452
City: Rotterdam
Type of rheumatic disease: Rheumatoid Arthritis.</td>
</tr>
</table>

**Re: Mijn man moet chemo...**<br><br>door **sylvia35** » vr aug 14, 2015 3:15 pm<br><br>Hallo Maria,<br><br>Bedankt voor uw verhalen en dat u het in deze moeilijke tijd me ons wil delen. Hoe gaat het nu met uw man? Heeft hij ook pijn in de botten?<br>Mag ik vragen hoe oud hij is? (Misschien staat het al in een van uw berichten dat ik het niet gezien heb).<br><br>Mijn man heeft uitzaaiingen in de botten en een agressieve vorm pk. Sinds december gaat het een stuk slechter met hem. Op het moment begint hij nu wel pijn in zijn botten te krijgen. Vandaag gaan we naar het ziekenhuis, ik zal eens vragen wat daar aan te doen is.<br><br>Ik hoop dat de chemo bij uw man goed aanslaat. Heel veel sterkte!	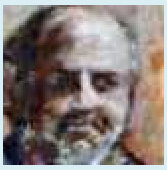<br>Silvia35<br>**Berichten:** 3<br>**Geregistreerd:** vr 20 mei 2011 15:19
[Translation]<br>**Re: My husband needs chemo therapy…**<br><br>**By sylvia35** » Friday Aug 14, 2015 3:15 PM<br><br>Hello Maria<br><br>Thanks for sharing your experiences in this difficult time. How is your husband currently doing? Does he have painful bones as well? Can I ask how old he is? (Maybe you already mentioned that in one of your messages and I didn't see it).<br><br>My husband has metastatsis in the bones and an aggressive form of pc. Since December he is worsening. At the moment he is starting to get sore bones. Today we are visiting the hospital, I will ask what can be done about that.<br><br>I hope your husband's chemotherapy will work. Wish you strength!	Sylvia35<br>**Messages**: 3<br>**Registered**: Fr May 20 2011 15:19

**Iemand ervaring met neuofeedback? ...**<br><br>door **Annelies van Tummeren** » do apr 9, 2015 7:32 pm<br><br>Hoi allemaal,<br><br>Onze oudste zoon, Dries, van 13 jaar heeft al vanaf zijn kleutertijd last van ADHD. We hebben 'm een tijdje Ritalin gegeven, maar overwegen nu om 'm op te geven voor neurofeedback-therapie. Dat kan hier in de stad (Eindhoven), maar is wel nogal duur. Heeft er iemand hier ervaring hiermee?<br>Groet, Annelies	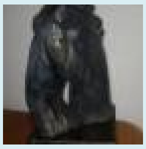<br>Annelies van Tummeren<br>**Berichten:** 2<br>**Geregistreerd:** za 12okt 2013 11:31
[Translation]<br><br>**Somebody who has experience with neuofeedback? …**<br><br>**By: Annelies van Tummeren** » Thursday Apr 9, 2015 7.32 PM	Annelies van Tummeren<br>**Messages**: 2<br>**Registered**: Sa Oct 12 2013 11:31

Hi everybody<br><br>Our oldest son, Dries, is 13 years old and has ADHD since he was a toddler. We have given him Ritalin for a while, but we are considering to sign him up for neurofeedback-therapy. This is available in our city (Eindhoven), but it is quite expensive. Does anyone has experience with this?<br><br>Greetings, Annelies	

Imagine, you leave for a vacation to Morocco in two weeks. You suddenly realize that you don’t know if you need any vaccinations for this trip. You decide to write your general practitioner an e-mail to ask him/her.

Write down below what you would ask your doctor in this situation:

……….

……….

………

………
